# PPARs and RXRs in Male and Female Fertility and Reproduction

**DOI:** 10.1155/2008/637490

**Published:** 2008-04-06

**Authors:** P. Froment

**Affiliations:** Unité de Physiologie de la Reproduction et des Comportements, UMR 6175 INRA-CNRS, Université F. Rabelais de Tours Haras Nationaux, 37380 Nouzilly, France

Peroxisome proliferator-activated receptors (PPARs) are ligand-activated nuclear receptors controlling many important physiological processes, including energy homeostasis, lipid, and glucose metabolism, inflammation, as well as cell proliferation and differentiation. PPARs and their heterodimeric partner (retinoid X receptor, RXR) have been found in early embryo, developing fetus, and in various compartments of the reproductive system (hypothalamus, pituitary gland, ovary, uterus, and testis) of many species (birds, fishes, mammals such as rodents, cattle, pigs, and humans) [1]. The reviews in this special issue paint a broad picture of the potential role of the PPAR-RXR system in the reproductive axis. They also raise new questions about the biological actions of the PPAR-RXR system in reproduction and its possible manipulation in treatments of fertility disorders (due to problems with energy metabolism, or specific diseases).

Over the last 10 years, a number of studies in vivo and in vitro have strongly suggested that these nuclear receptors might play an important role from gametogenesis to parturition, including gestation and the links mother/fetus [2]. Thus, PPARs are expressed in the testis where lipid metabolism and specially the *β*-oxidation of fatty acids are important for testicular functions (steroids synthesis, lipid composition of the sperm, etc.). In addition, some testicular toxicants such as phthalates bind to PPAR*α* and PPAR*γ* and modify their activities. In female, invalidation of PPAR*γ* in the mouse ovary [3] leads to a decrease in fecundity probably due to a drop in the production of sexual steroids. Mice null for PPAR*β*/*δ*, PPAR*γ*, or RXR*α* [4–6] display alterations in the attachment of embryos to the endometrium and/or placenta development and function. During the labor, mRNA expression of cyclooxygenase-2, an inducer of contractions of the myometrium and a PPAR target gene, is increased in fetal membranes when the PPAR*α* and *γ* expression drops at the start of parturition. After birth, PPARs continue to play a role in the relation mother/neonates through the mammary gland functions. Indeed, in transgenic mice, a constitutive activation of PPAR*α* in mammary gland alters its development and the lactation leading to mortality of neonates [7]. Furthermore, mice with a deletion of PPAR*γ* in mammary gland produce a “toxic milk” containing elevated levels of inflammatory lipids. Despite these strong phenotypes, the mechanisms of action of these receptors remain unclear in the control of fertility and further investigations are needed to better use them in medical treatments.

In the future, these drugs might also be used in a large spectrum of treatments targeting reproduction such as improvement of follicular development, in vitro fertilization, certain complications of pregnancy, and sex hormone-sensitive cancers affecting the reproductive tissues, including breast, prostate, ovary, or cancers affecting pituitary cells (pituitary adenomas). For example, new generation of pharmacological drugs targeting these receptors are already in clinical use or are undergoing testing for use as therapeutic agents. Synthetic molecules (glitazone molecules, which bind to PPAR*α*, or glitazar molecules, which bind to PPAR*α*/PPAR*γ*), currently being tested in clinical studies, may prove particularly useful for the treatment of certain types of infertility associated with metabolism disorders such as insulinoresistance in polycystic ovary syndrome (PCOS) [8]. The therapeutical treatment in women with pregnancy-specific diseases could be also changed. Women with severe preeclampsia have a reduction in serum levels of PPAR activating lipids several weeks before the onset of symptoms. The use of PPAR ligands might be proposed to ameliorate the disorders associated with preeclampsia such as hypertension and inflammation. Moreover, the rate of success of in vitro fertilization (IVF) could be improved in the next decade by addition in the culture media of PPAR*β*/*δ* ligands. Recent studies have shown that the development and implantation of IVF embryos may be augmented by supplementing culture media with PGI2 analogs, a synthetic PPAR*β*/*δ* ligand, or retinoic acid [9]. Of note, in all these examples, potential long-term adverse effects are unknown, and more data need to be acquired to consider these drugs “safe” during pregnancy.

Finally, in this issue, we report the putative functions of PPARs and RXRs in gonads, placenta, and embryo and we will discuss the possible role of PPARs as mediators of environmental toxicity for reproductive function.

*P. Froment* *P. Froment*
